# Qual a Eficácia do Índice Imune Inflamatório Sistêmico na Etiopatogenia da Ectasia Isolada da Artéria Coronária?

**DOI:** 10.36660/abc.20230048

**Published:** 2023-08-18

**Authors:** Kenan Toprak, Mustafa Kaplangoray, Mehmet Inanır, Tolga Memioğlu

**Affiliations:** 1 Harran University Department of Cardiology Sanliurfa Turquia Harran University - Department of Cardiology, Sanliurfa – Turquia; 2 Bilecik Şeyh Edebali Üniversitesi – Cardiology Bilecik Turquia Bilecik Şeyh Edebali Üniversitesi – Cardiology, Bilecik – Turquia; 3 Abant Izzet Baysal University Cardiology Department Bolu Turquia Abant Izzet Baysal University - Cardiology Department, Bolu – Turquia

**Keywords:** Doença Arterial Coronariana/complicações, Dilatação Patológica Biomarcadores Inflamação Imune Sistêmica

A ectasia isolada da artéria coronária (EIAC) é frequentemente encontrada na prática clínica com métodos de imagem invasivos crescentes, e nosso conhecimento sobre sua etiologia, prognóstico e abordagens de tratamento está aumentando diariamente.^1,2^ Lemos com grande interesse o recente estudo retrospectivo de Dindas et al.,^3^ que trata da relação entre o Índice de Inflamação Imunológica Sistêmica (IIS) e EIAC.^3^ Foi sugerido que neutrófilos, plaquetas e linfócitos IIS coletados em uma única fração podem ser um bom indicador de inflamação e resposta imune.^4^ Vários estudos mostraram que o IIS pode ter um valor prognóstico mais potente do que os marcadores inflamatórios convencionais, como Relação Neutrófilos/Linfócitos (RNL) e Relação Plaquetas/Linfócitos (RPL).^5^ Gostaríamos de comentar sobre o artigo bem elaborado e apresentado,que pensamos contribuir significativamente para a literatura.

Primeiramente, na análise da curva Receiver Operating Characteristic (ROC) do artigo de Dindas et al.,^3^ concluiu-se que IIS previu pacientes com EIAC melhor do que RNL, RPL e Relação Monócitos/HDL (RMH).^3^ Na análise da curva ROC do artigo de Dindas et al.,^3^ observou-se que IIS tinha maior AUC do que RNL, RPL e RMH, e a partir desses resultados, concluiu-se que IIS previu pacientes isolados com EC melhor do que RNL, RPL e RMH. No entanto, nenhuma comparação estatisticamente pareada foi feita entre as curvas ROC. Portanto, é difícil olhar apenas para os valores numéricos dos valores AUC e dizer que IIS é um preditor EIAC mais forte e valioso do que RNL, RPL e RMH. Na verdade, uma vez que a AUC de SII está próxima da AUC de RNL (0,832 vs. 0,780), uma comparação estatística pareada entre SII e RNL é essencial.^6,7^

Em segundo lugar, pacientes com EIAC, DAC obstrutiva sem ectasia coronariana e pacientes com artérias coronárias angiograficamente normais foram comparados no estudo para formar três grupos. No entanto, sabe-se que a ectasia coronariana é considerada uma variante da doença arterial coronariana e muitas vezes é detectada concomitantemente à DAC obstrutiva, em vez de ser isolada.^8,9^ Na verdade, a classificação de Markis não é uma classificação usada apenas para coronariopatia isolada ectasia arterial.^9^ Nesse contexto, pensamos que a inclusão de pacientes com DAC obstrutiva acompanhada de ectasia coronariana no estudo pode revelar mais claramente o efeito do IIS no EIAC.

## References

[1] Toprak K, Kaplangoray M, Palice A (2022). The Impact of C-Peptide and Diabetes Mellitus on Coronary Ectasia and Effect of Coronary Ectasia and C-Peptide on Long-Term Outcomes: A Retrospective Cohort Study. Int J Clin Pract.

[2] Toprak K, Kaplangoray M, Altiparmak İ.H, Taşcanov M. B, Güngören F, Fedai H (2022). Can increased intestinal permeability and low-grade endotoxemia be the triggering pathogenesis in isolated coronary artery ectasia?. Coronary Artery Disease.

[3] Dindas F, Koyun E, Turkyilmaz E, Abacioglu OO, Yildirim A, Sahin A (2023). Systemic Immune Inflammation Index is a Novel Marker in Predicting the Presence and Severity of Isolated Coronary Artery Ectasia. Arq Bras Cardiol.

[4] Hu B, Yang XR, Xu Y, Sun YF, Guo W (2014). Systemic immune-inflammation index predicts prognosis of patients after curative resection for hepatocellular carcinoma. Clin Cancer Res.

[5] Gao Y, Guo W, Cai S, Zhang F, Shao F, Zhang G (2019). Systemic immuneinflammation index (SII) is useful to predict survival outcomes in patients with surgically resected esophageal squamous cell carcinoma. J Cancer.

[6] Metz EC, Herman BA, Roe CA (1998). Statistical comparison of two ROC-curve estimates obtained from partially-paired datasets. Med Decis Making.

[7] Vergara IA, Norambuena T, Ferrada E, Slater AW, Melo F (2008). StAR: a simple tool for the statistical comparison of ROC curves. BMC Bioinformatics.

[8] Devabhaktuni S, Mercedes A, Diep J, Ahsan C (2016). Coronary Artery Ectasia-A Review of Current Literature. Curr Cardiol Rev.

[9] Zhang Y, Huang QJ, Li XL, Guo YL, Zhu CG, Wang XW (2015). Prognostic Value of Coronary Artery Stenoses, Markis Class, and Ectasia Ratio in Patients with Coronary Artery Ectasia. Cardiology.

