# Supplementary material for: Qual a Eficácia do Índice Imune Inflamatório Sistêmico na Etiopatogenia da Ectasia Isolada da Artéria Coronária?
Source: Arq Bras Cardiol. 2023 Aug 18;120(7):e20230048. [Article in Portuguese] doi: 10.36660/abc.20230048 (PMC10519350; doi:10.36660/abc.20230048)
Supplement: Translation

# Translation: How Effective is the Systemic Inflammatory Immune Index in the Etiopathogenesis of Isolated Coronary Artery Ectasia?

**Authors:** Kenan Toprak, Mustafa Kaplangoray, Mehmet Inanır, Tolga Memioğlu

**Affiliations:** 1 Sanliurfa Turkey Harran University - Department of Cardiology, Sanliurfa – Turkey; 2 Bilecik Turkey Bilecik Şeyh Edebali Üniversitesi – Cardiology, Bilecik – Turkey; 3 Bolu Turkey Abant Izzet Baysal University - Cardiology Department, Bolu – Turkey

**Keywords:** Coronary Artery Disease/complications, Dilatation Pathologic, Sistemic, Inflammation Immune

Isolated coronary artery ectasia (ICAE) is frequently encountered in clinical practice with increasing invasive imaging methods, and our knowledge about its etiology, prognosis, and treatment approaches is increasing daily.^1,2^ We read with great interest the recent retrospective study of Dindas et al.,^3^ which deals with the relationship between the Systemic Immune Inflammation Index (SII) and ICAE.^3^ It has been suggested that SII neutrophils, platelets, and lymphocytes collected in a single fraction may be a good indicator of inflammation and immune response.^4^ Various studies have shown that SII may have a more potent prognostic value than conventional inflammatory markers such as Neutrophil Lymphocyte Ratio (NLR) and Platelet Lymphocyte Ratio (PLR).^5^ We would like to comment on the well-designed and presented article, which we think will contribute significantly to the literature.

Firstly, in the Receiver Operating Characteristic (ROC) curve analysis of Dindas et al.^3^ article, it was seen that SII had a higher area under the curve (AUC) than NLR, PLR, and MHR, and from these results, it was concluded that SII predicted ICAE patients better than NLR, PLR and Monocyte HDL Ratio (MHR).^3^ In the ROC curve analysis of Dindas et al.^3^ article, it was seen that SII had higher AUC than NLR, PLR, and MHR, and from these results, it was concluded that SII predicted isolated CE patients better than NLR, PLR, and MHR. However, no statistically pairwise comparison was made between ROC curves. Therefore, it is difficult to look at the numerical values of the AUC values alone and say that SII is a stronger and more valuable ICAE predictor than NLR, PLR, and MHR. As a matter of fact, since the AUC of SII is close to the AUC of NLR (0.832 vs. 0.780), a pairwise statistical comparison between SII and NLR is essential.^6,7^

Secondly, patients with ICAE, obstructive CAD without coronary ectasia, and patients with angiographically normal coronary arteries were compared in the study to form three groups. However, it is known that coronary ectasia is considered a variant of coronary artery disease and is often detected concomitantly with obstructive CAD rather than being isolated.^8,9^ As a matter of fact, the Markis classification is not a classification used only for isolated coronary artery ectasia.^9^ In this context, we think that including patients with obstructive CAD accompanied by coronary ectasia in the study may reveal the effect of SII on ICAE more clearly.
