# Supplementary material for: Qual a Eficácia do Índice Imune Inflamatório Sistêmico na Etiopatogenia da Ectasia Isolada da Artéria Coronária?
Source: Arq Bras Cardiol. 2023 Aug 18;120(7):e20230048. [Article in Portuguese] doi: 10.36660/abc.20230048 (PMC10519350; doi:10.36660/abc.20230048)
Supplement: Related texts

# Reply: Carta-resposta

**Authors:** Ferhat Dindaş, Emin Koyun, Erdem Türkyilmaz, Ozge Ozcan Abacioglu, Arafat Yıldırım, Anil Sahin, Barış Dindar, Mustafa Dogdus, Ozkan Candan

**Para o editor,**

Estamos satisfeitos que os autores tenham lido nosso artigo com grande interesse. De fato, como afirmado na introdução e no título de nosso estudo, objetivamos investigar a possível relação entre ectasia coronariana (EC) isolada e um novo parâmetro de inflamação, o Índice de Inflamação Imune Sistêmica (IIS), em pacientes com angina pectoris estável ou instável.^1^ Obtivemos resultados sugerindo que o IIS pode ser um índice mais eficaz do que outros parâmetros inflamatórios hematológicos na distinção de pacientes com EC isolada.^1^ Uma comparação estatística pareada não foi incluída em nosso estudo porque pode estar fora do objetivo principal do estudo. No entanto, existem exemplos de artigos na literatura atual que não foram comparados com comparações pareadas com valores de corte próximos na análise ROC de parâmetros numéricos.^2,3^ Além disso, muitos estudos separados na literatura mostram que o IIS tem um valor preditivo mais forte do que a Razão Neutrófilo/Linfócito (RNL) em pacientes com doenças cardiovasculares.^4–6^

O autor tem razão ao enfatizar que o isolamento da EC não deve constituir um conceito diferente de doença arterial coronariana. Enquanto a EC isolada foi definida como a ausência de evidência de estenose coronariana acompanhando a ectasia em estudos anteriores,^7^ a EC isolada foi definida em estudos recentes como a ausência de uma lesão que cause mais de 50% de obstrução acompanhando a ectasia.^3,8^ À luz dessa informação, nosso estudo tentou mostrar o efeito inflamatório óbvio da IIS examinando o grupo de pacientes com estenose da artéria coronária epicárdica menor que 50% acompanhada de ectasia no grupo EC isolado.^1^ No entanto, avaliar pacientes com EC de tipo misto com mais de 50% de obstrução será muito impressionante como para ser ignorado em estudos futuros.

## References

[1] Dindas F, Koyun E, Turkyilmaz E, Abacioglu OO, Yildirim A, Sahin A (2023). Systemic Immune Inflammation Index is a Novel Marker in Predicting the Presence and Severity of Isolated Coronary Artery Ectasia. Arq Bras Cardiol.

[2] Huang H, Liu Q, Zhu L, Zhang Y, Lu X, Wu Y, Liu L (2019). Prognostic Value of Preoperative Systemic Immune-Inflammation Index in Patients with Cervical Cancer. Sci Rep..

[3] Dereli S, Çerik İB, Kaya A, Bektaş O (2020). Assessment of the Relationship Between C-Reactive Protein-to-Albumin Ratio and the Presence and Severity of Isolated Coronary Artery Ectasia. Angiology.

[4] Candemir M, Kiziltunç E, Nurkoç S, Şahinarslan A (2021). Relationship Between Systemic Immune-Inflammation Index (SII) and the Severity of Stable Coronary Artery Disease. Angiology.

[5] Erdoğan M, Erdöl MA, Öztürk S, Durmaz T (2020). Systemic immune-inflammation index is a novel marker to predict functionally significant coronary artery stenosis. Biomark Med.

[6] Kelesoglu S, Yilmaz Y, Elcık D, Kalay N (2022). Systemic immune inflammation index: a novel predictor for coronary collateral circulation. Perfusion.

[7] Zhang Y, Huang QJ, Li XL, Guo YL, Zhu CG, Wang XW, Xu B, Gao RL, Li JJ (2015). Prognostic Value of Coronary Artery Stenoses, Markis Class, and Ectasia Ratio in Patients with Coronary Artery Ectasia. Cardiology.

[8] Özkan B, Örsçelik Ö, Yıldırım Yaroğlu H, Balcı Ş, Özcan MK, Çelik A, Özcan IT (2019). Association between serum adropin levels and isolated coronary artery ectasia in patients with stable angina pectoris. Anatol J Cardiol.

# Reply

**Authors:** Ferhat Dindaş, Emin Koyun, Erdem Türkyilmaz, Ozge Ozcan Abacioglu, Arafat Yıldırım, Anil Sahin, Barış Dindar, Mustafa Dogdus, Ozkan Candan

**To the Editor,**

We are pleased that the authors read our article with great interest. In fact, as stated in the introduction and title of our study, we aimed to investigate the possible relationship between isolated coronary ectasia (CE) and a new inflammation parameter, Systemic Immune Inflammation Index (SII), in patients with stable or unstable angina pectoris.^1^ We obtained results suggesting that SII may be a more effective index than other hematological inflammatory parameters in distinguishing patients with isolated CE.^1^ A pairwise statistical comparison was not included in our study because it may be outside the study's main purpose. However, there are examples of articles in the current literature that have not been compared with pairwise comparisons with close cut-off values in ROC analysis of numerical parameters.^2,3^ In addition, many separate studies in the literature show that SII has a stronger predictive value than Neutrophil to Lymphocyte Ratio (NLR) in patients with cardiovascular diseases.^4–6^

The author is right in emphasizing that the isolation of CE should not constitute a concept other than coronary artery disease. While isolated CE was defined as the absence of evidence of coronary stenosis accompanying ectasia in previous studies,^7^ isolated CE has been defined in recent studies as the absence of a lesion that causes more than 50% obstruction accompanying ectasia.^3,8^ In light of this information, our study tried to show the obvious inflammatory effect of SII by examining the patient group with epicardial coronary artery stenosis of less than 50% accompanied by ectasia in the isolated CE group.^1^ However, evaluating patients with mixed-type CE with more than 50% obstruction will be too impressive to ignore in future studies.
